# Developmental changes in independent bimanual coordination: evidence from the circles–lines coupling task in children aged 5–13 years

**DOI:** 10.3389/fnhum.2025.1620941

**Published:** 2025-07-24

**Authors:** Satoshi Nobusako, Kenya Hashizoe, Akio Nakai

**Affiliations:** ^1^Neurorehabilitation Research Center, Kio University, Nara, Japan; ^2^Graduate School of Health Science, Kio University, Nara, Japan; ^3^Department of Rehabilitation, Yamada Hospital, Gifu, Japan; ^4^Research Institute for Education and Graduate School of Clinical Education, Mukogawa Women’s University, Nishinomiya, Japan

**Keywords:** bimanual coordination, children, circles-lines coupling task, fine motor skills, manual dexterity, motor development

## Abstract

**Introduction:**

Bimanual coordination, particularly the ability to perform independent and simultaneous asymmetric movements with both hands, is essential for many daily activities and develops throughout childhood. However, its developmental trajectory remains unclear. This study investigated age-related changes in bimanual coordination using the bimanual circles–lines coupling (BC) task and explored its relationship with fine motor skills in children aged 5 to 13 years.

**Methods:**

A total of 150 typically developing children completed the BC task under unimanual (UM) and bimanual (BM) conditions. The bimanual coupling effect (BCE), reflecting interference between concurrent motor programs, was calculated as the difference in ovalization index (OI) between the two conditions. Fine motor abilities were assessed using the manual dexterity component of the Movement Assessment Battery for Children–2nd Edition (M-ABC2), yielding scores for preferred hand, non-preferred hand, bimanual, and handwriting skills.

**Results:**

Across age groups, OI was significantly higher in the BM than in the UM condition, indicating the presence of BCE. Both UM-OI and BM-OI significantly decreased with age, while BCE showed a weak but significant negative correlation with age. These findings suggest a gradual developmental improvement in the ability to perform independent bimanual movements. Partial correlation analyses, controlling for age, revealed significant associations between BCE/BM-OI and bimanual motor skills, suggesting that the ability to overcome intermanual interference is closely linked to age-appropriate bimanual coordination.

**Discussion:**

Given that asymmetric bimanual control involves not only the fronto-parietal network but also executive functions in the prefrontal cortex and interhemispheric motor communication, the present behavioral findings may reflect multifaceted neural maturation during childhood—specifically, the development of the fronto-parietal network, prefrontal cortex, and transcallosal motor pathways. The BC task, which requires only minimal equipment and administration time, may serve as a valid, cost-effective, and developmentally sensitive tool for quantifying bimanual coordination abilities in children. Its practical applicability highlights its potential as an assessment measure for motor development in both research and clinical contexts. This study contributes to a better understanding of how independent bimanual control emerges during childhood and may inform future approaches to evaluating fine motor function in pediatric neuromotor disorders.

## Highlights

- Independent bimanual coordination improves gradually from ages 5 to 13, as assessed by the bimanual circles–lines coupling (BC) task.
- The bimanual coupling effect (BCE) shows a weak but significant negative correlation with age in typically developing children.
- Bimanual coordination performance is significantly associated with age-adjusted bimanual motor skills measured by the M-ABC2.
- The BC task is a valid and practical tool for assessing developmental changes in bimanual coordination across childhood.

## Introduction

Bimanual coordination skills are essential for countless everyday activities such as typing, meal preparation, and driving (Gooijers and Swinnen, 2014). Many of these daily tasks require not symmetrical but simultaneous and asymmetric use of both hands. It is well established that performing asymmetric bimanual movements often results in interference effects, where the motor programs for each hand influence each other (Chan and Chan, 1995). This intermanual interference is considered to reflect interactions between two distinct motor programs or between perceptual goals (Heuer, 1993; Mechsner et al., 2001; Wolpert and Ghahramani, 2000).

A well-known paradigm for quantitatively measuring this interference is the bimanual circles–lines coupling (BC) task (Franz et al., 1991; Franz, 1997; Garbarini et al., 2012, 2013, 2014). In this task, participants are asked to draw circles and vertical lines simultaneously under two experimental conditions. In the unimanual (UM) condition, participants repeatedly draw vertical lines with their preferred hand. In the bimanual (BM) condition, participants simultaneously draw circles with their non-preferred hand while continuing to draw vertical lines with their preferred hand. Typically, the vertical lines drawn in the BM condition are distorted into elliptical shapes compared to those in the UM condition, due to the interference from the circular motor program. The magnitude of this interference is referred to as the bimanual coupling effect (BCE), calculated by subtracting the ovalization index (OI) in the UM condition from that in the BM condition (Garbarini et al., 2012, 2013, 2014; Piedimonte et al., 2014, 2018). A lower BCE indicates a higher ability to independently control each hand during simultaneous movement (Piedimonte et al., 2014).

However, the developmental trajectory of the BCE remains largely unclear. To date, only one study has investigated the age-related changes in BCE using the BC task. Piedimonte et al. (2014) examined BCE across four age groups—children aged 6 and 10 years, young adults aged 20–30 years, and older adults aged 60–80 years—and found that the BCE was highest in the 6- and 10-year-old children and lowest in the young adult group. These findings suggest that the ability to independently control both hands simultaneously improves with age, but the developmental progression during childhood remains to be clarified.

Several neuroscientific studies suggest that BCE relies on the activity of the fronto-parietal network. Garbarini et al. (2014) reported that during the BM condition of the BC task (i.e., one hand drawing lines, the other drawing circles), the supplementary motor area (SMA) and posterior parietal cortex (PPC)—key regions of the fronto-parietal network—showed greater activation compared to symmetrical bimanual movements (e.g., both hands drawing lines). Similarly, Iester et al. (2025) demonstrated that the BM condition elicited stronger activation in the primary sensorimotor cortex (Brodmann areas [BA] 3 and 4), premotor cortex (BA6), superior parietal lobule (BA7), and inferior parietal lobule (BA39, 40) than symmetrical conditions. These authors proposed that the BM condition involves greater engagement of both sensorimotor and associative areas to manage the competition and coordination between two independent motor programs (Iester et al., 2025). In particular, the inferior parietal lobule is thought to play a central role in sensorimotor integration, behavioral monitoring, and intermanual information mediation (Iester et al., 2025).

Furthermore, while the primary sensorimotor cortex matures relatively early, by around age six, the fronto-parietal network—including the prefrontal cortex and parietal associative areas—undergoes gradual maturation during adolescence (Casey et al., 2005). Gogtay et al. (2004) similarly reported a sequential maturation pattern from sensory-motor regions to parietal association cortices and, finally, to the dorsolateral prefrontal cortex from age 4 to 16. These findings suggest that the BCE, reflecting the ability to independently control each hand, is likely to undergo developmental changes during childhood. Nevertheless, its precise developmental course remains unclear.

Additionally, because the BC task assesses the ability to perform independent simultaneous hand movements, it is expected to relate to upper-limb motor abilities, particularly fine motor skills. However, it remains unknown which specific fine motor skills are associated with BCE. A widely used standardized battery to assess children’s coordination abilities is the Movement Assessment Battery for Children–2nd edition (M-ABC2; Henderson et al., 2007). The manual dexterity component of M-ABC2 provides age-adjusted quantitative evaluations of four fine motor skills: preferred-hand skill, non-preferred-hand skill, bimanual skill, and handwriting skill.

Therefore, in the present study, we administered the BC task to children aged 5 to 13 years to investigate the developmental changes in their ability to perform independent simultaneous bimanual movements. We also evaluated their fine motor skills using the M-ABC2 and examined the relationship between BCE and each type of fine motor skill.

## Materials and methods

### Participants

A total of 150 typically developing children (mean age ± standard deviation, 8.9 ± 2.4 years; range, 5–13 years; 71 male; 139 right-handed) participated in the study. The participants were recruited from regular classes at nursery schools, primary schools, and secondary schools in Osaka and Nara, Japan. Based on age, they were divided into nine age groups ranging from 5 to 13 years old (Table 1).

**Table 1:** Summary of data for each age group.

Group		Sex	Preferred hand	Fine motor skills					Bimanual coupling task		
				Preferred hand skill	Non-preferred hand skill	Bimanual skill	Handwriting skill	Total	UM-OI	BM-OI	BCE
5-year-old (*n* = 9)	Mean	Male = 5 Female = 4	Right = 9 Left = 0	8.1	10.2	8.9	11.2	10.4	12.961	26.891	13.930
	SD			4.3	3.5	4.3	0.6	2.9	8.444	17.608	21.198
	Max			13	13	14	13	14	29.007	71.946	67.996
	Min			1	1	1	11	6	3.950	14.114	−9.872
	Skewness			−0.641	−1.850	−0.433	2.475	−0.038	0.852	1.746	1.646
	Kurtosis			−0.825	5.601	−1.068	9.000	−1.909	−0.129	4.692	4.813
6-year-old (*n* = 21)	Mean	Male = 9 Female = 12	Right = 18 Left = 3	10.6	10.7	11.9	10.9	12.2	8.360	18.577	10.217
	SD			2.9	3.0	3.0	0.4	2.2	3.216	8.567	9.540
	Max			15	17	15	11	15	18.063	45.150	39.108
	Min			5	5	3	9	6	5.199	6.693	−11.370
	Skewness			−0.423	−0.201	−1.449	−4.249	−0.820	1.630	1.635	0.942
	Kurtosis			−0.315	−0.239	2.644	21.000	1.819	2.871	3.584	3.941
7-year-old (*n* = 19)	Mean	Male = 7 Female = 12	Right = 16 Left = 3	13.1	11.8	10.5	11.2	12.6	7.465	17.178	9.712
	SD			2.3	1.9	3.1	2.5	2.6	2.915	6.384	6.490
	Max			15	15	15	12	17	14.341	33.153	21.343
	Min			7	7	3	1	6	3.992	7.842	−2.904
	Skewness			−1.393	−0.884	−0.768	−3.644	−0.693	0.990	0.797	−0.047
	Kurtosis			1.840	1.158	0.181	16.359	0.750	0.228	0.440	−0.971
8-year-old (*n* = 24)	Mean	Male = 11 Female = 13	Right = 22 Left = 2	10.5	12.0	10.7	11.1	11.9	7.121	15.526	8.404
	SD			2.2	2.1	2.6	2.4	2.3	3.740	5.718	5.800
	Max			16	16	15	12	16	19.497	25.175	21.274
	Min			7	8	4	4	7	2.583	6.849	1.564
	Skewness			0.243	0.224	−0.968	−2.290	−0.203	1.626	0.030	0.580
	Kurtosis			0.652	−0.961	0.753	4.392	−0.242	3.814	−1.292	−0.707
9-year-old (*n* = 18)	Mean	Male = 11 Female = 7	Right = 18 Left = 0	12.9	12.3	11.1	10.7	12.7	5.892	11.756	5.864
	SD			2.2	2.5	2.0	1.1	2.2	2.112	4.436	4.348
	Max			16	16	15	11	16	12.086	21.081	14.394
	Min			9	9	7	6	8	3.190	3.880	−1.827
	Skewness			−0.280	0.056	0.292	−3.881	−0.125	1.272	0.144	0.169
	Kurtosis			−0.840	−1.469	−0.325	18.000	−0.408	2.569	−0.537	−0.545
10-year-old (*n* = 15)	Mean	Male = 7 Female = 8	Right = 15 Left = 0	9.8	11.3	9.9	10.2	11.2	4.735	13.113	8.378
	SD			3.7	3.4	2.9	2.1	3.0	1.263	4.823	4.112
	Max			14	16	15	11	15	6.893	22.315	15.667
	Min			3	5	6	4	6	3.040	5.932	2.892
	Skewness			−0.541	−0.405	0.194	−2.291	−0.313	0.539	0.408	0.404
	Kurtosis			−0.801	−0.728	−1.697	5.548	−1.180	−0.970	−0.722	−1.073
11-year-old (*n* = 17)	Mean	Male = 9 Female = 8	Right = 16 Left = 1	10.8	12.2	10.5	12.2	12.7	4.809	11.936	7.127
	SD			2.5	1.7	2.7	1.3	2.5	2.855	5.477	3.748
	Max			16	16	15	13	19	13.925	25.008	15.621
	Min			5	10	4	10	9	1.896	5.623	1.387
	Skewness			−0.148	0.761	−0.708	−0.982	0.974	1.979	1.027	0.796
	Kurtosis			0.764	0.140	1.034	−0.862	0.876	5.496	0.487	0.937
12-year-old (*n* = 12)	Mean	Male = 7 Female = 5	Right = 10 Left = 2	12.0	11.6	11.3	12.3	13.4	4.863	10.952	6.089
	SD			1.9	2.3	2.7	1.5	2.8	1.908	4.234	4.214
	Max			16	14	14	13	18	10.106	19.199	13.743
	Min			9	6	5	9	9	2.451	6.528	−1.468
	Skewness			0.356	−1.024	−0.924	−1.789	−0.006	1.405	0.638	0.112
	Kurtosis			−0.173	1.425	0.914	2.640	−0.987	4.099	−0.964	−0.514
13-year-old (*n* = 15)	Mean	Male = 5 Female = 10	Right = 15 Left = 0	10.4	10.1	8.0	11.5	10.3	4.804	11.857	7.053
	SD			3.2	2.6	4.1	1.9	2.3	2.163	6.605	6.147
	Max			15	13	15	13	15	8.412	29.336	24.343
	Min			5	4	1	6	6	2.191	3.837	0.604
	Skewness			−0.243	−0.782	−0.180	−1.748	0.097	0.447	0.990	1.360
	Kurtosis			−1.157	0.204	−0.570	3.615	0.093	−1.183	1.568	2.757

BCE, bimanual coupling effect; BM-OI, the ovalization index (OI) in the bimanual condition (BM); Max, maximum; Min, minimum; SD, standard deviation; UM-OI, the ovalization index (OI) in the unimanual condition (UM).

The exclusion criteria were: (1) a general medical condition, e.g., cerebral palsy, hemiplegia, and muscular dystrophy; (2) diagnosis of a developmental disorder, e.g., autism spectrum disorder, attention deficit hyperactivity disorder, developmental coordination disorder, and learning disorder; or (3) diagnosis of intellectual disability. Eligibility was confirmed by interviewing parents and the results of regular checkups, which were provided by the doctor at each school. All experimental procedures were approved by the local ethics committee of the institution to which the authors belongs (approval number: R4-41). There were no foreseeable risks, and no personally identifying information was collected. The participants and their parents provided background information and written informed consent. The procedures complied with the ethical standards of the 1964 Declaration of Helsinki regarding the treatment of human participants in research.

### Procedures

The participants completed two experimental tasks: the bimanual coupling (BC) task and the fine motor skills test of the Movement Assessment Battery for Children-2nd edition (M-ABC2). The two tasks were administered to each participant in a randomized order. The time required to complete each task was less than 20 min; all participants completed the two experimental tasks within 40 min.

### Bimanual circles-lines coupling (BC) task

**Figure 1:**
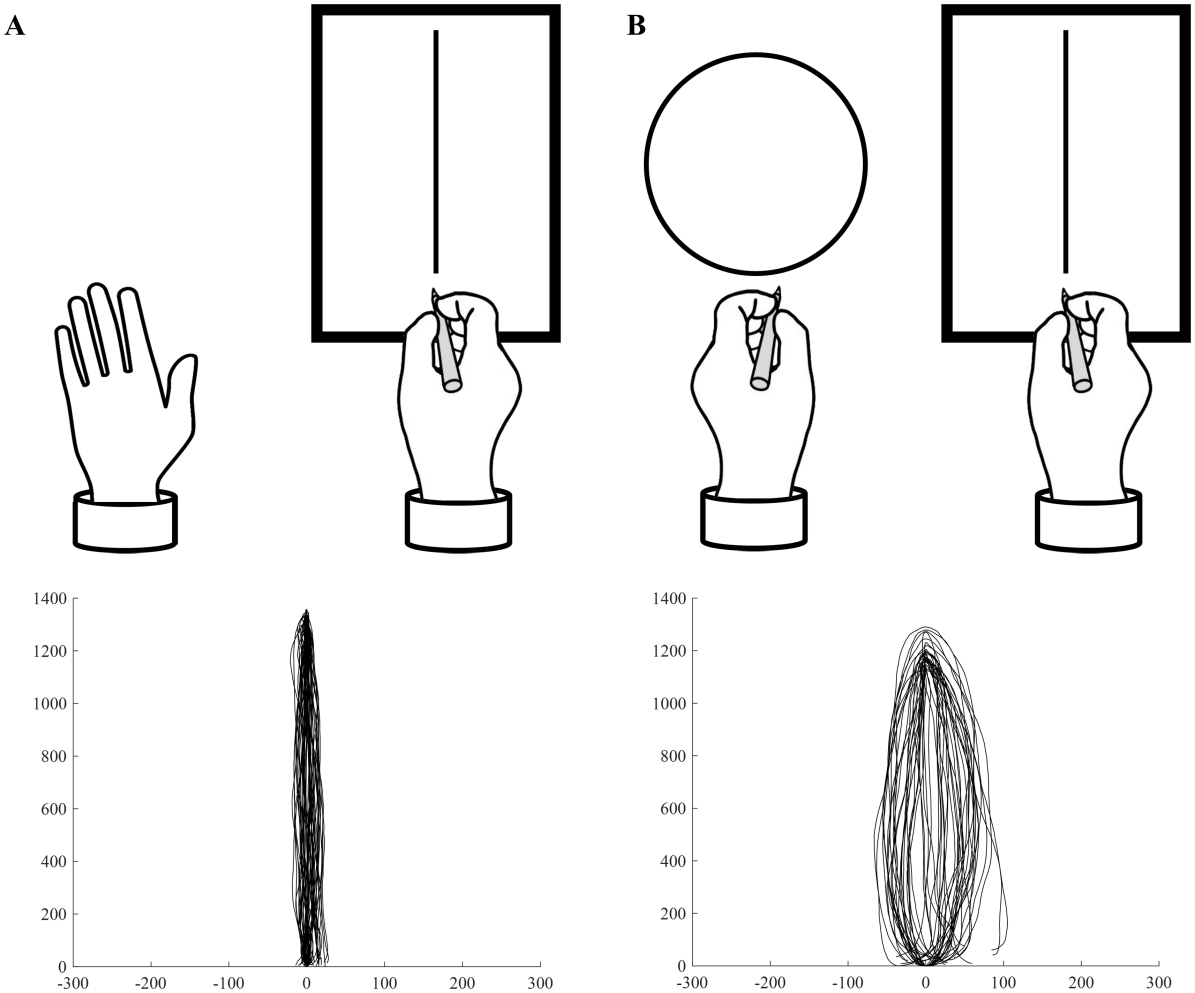
Bimanual circles-lines coupling task and resulting movement trajectories. **(A)** Unimanual condition. The participant repeatedly drew vertical straight lines on a tablet PC using their preferred hand. The lower panel shows an example from an 8-year-old right-handed female participant in the present study. **(B)** Bimanual condition. The participant repeatedly drew vertical straight lines on a tablet PC using their preferred hand while simultaneously drawing circles with their non-preferred hand. The lower panel shows an example from an 8-year-old right-handed female participant in the present study.

The BC task was conducted using a 13-inch tablet PC (width 162 mm × height 287 mm, resolution 2,880 × 1,920 pixels). The participants sat in a chair, with a tablet PC positioned on the preferred hand side of their body midline and an A4-sized blank sheet of paper (width 210 mm × height 297 mm) placed on the non-preferred hand side of the desk. This task had the following two conditions (Figure 1): (1) Unimanual (UM) condition: The participants repeatedly drew vertical lines with their preferred hand on the tablet PC. (2) Bimanual (BM) condition: The participants repeatedly drew circles with their non-preferred hand on the blank sheet while simultaneously drawing vertical lines with their preferred hand on the tablet PC. In all conditions, the starting posture was with the preferred hand holding the stylus in contact with the tablet PC on the desk. In each condition, after a verbal cue from the experimenter, the participants began drawing on the tablet PC (and on the blank sheet in the BM condition). In all conditions, the participants repeatedly drew straight lines with their preferred hand on the tablet PC for 30 s, and the trajectory of their movements was recorded. In the UM condition, the participants placed their non-preferred hand on the desk and did not move it. In the BM condition, the participants also held a pen in their non-preferred hand and repeatedly drew circles on the blank sheet. There were no restrictions on the speed or size of the lines and circles. In both conditions, participants were instructed and trained to move both hands in an in-phase manner—that is, when the preferred hand was positioned proximally toward the body, the non-preferred hand was also to be positioned proximally, and when the preferred hand moved distally, the non-preferred hand was to move distally at the same time. For all conditions, there was one practice trial before the actual task. Each participant performed the actual task once for each of the two conditions, with a 1-min break between conditions.

To quantify the distortion of the lines drawn with the preferred hand, we calculated the ovalization index (OI, %) for each condition (Garbarini et al., 2012, 2013, 2014). From the recorded trajectories in each trial, we identified the apical endpoints of the back-and-forth movements and extracted circular trajectories accordingly. For each circular trajectory, the long (y) and short (x) axes were determined using the following procedure, and the OI was calculated using the following formula:


$$\text{Variable}=\frac{\text{Standard deviation of short}\;(x)\;\text{axis data}}{\text{Standard deviation of long}\;(y)\;\text{axis data}}\times 100$$

For each participant, the OI was defined as the mean value of the variables obtained from all recorded cycle trajectories. An OI value close to 0 indicated minimal distortion of the trajectory toward a circular transformation (i.e., the trajectory remained nearly straight), whereas an OI value close to 100 indicated that the trajectory had fully transformed into a perfect circle. Furthermore, following previous studies (Garbarini et al., 2012, 2013, 2014), we calculated the bimanual coupling effect (BCE) by subtracting the OI value of UM condition (UM-OI) from the OI value of BM condition (BM-OI).

### Manual dexterity test of the M-ABC2

The manual dexterity test of the M-ABC2 (Henderson et al., 2007) is a standardized, age-adjusted test to identify motor problems in children, in which different tasks are administered according to age. The M-ABC2 has good test–retest reliability with a minimum value at any age of 0.75, inter-rater value of 0.70, and concurrent validity (Henderson et al., 2007). This test has three age bands: 3–6, 7–10, and 11–16 years.

The 5–6-year-old children were administered the posting coins (preferred and non-preferred hand), threading beads, and drawing trail I tests. The 7–10-year-old children were administered the placing pegs (preferred and non-preferred hand), threading lace, and drawing trail II tests. The 11–13-year-old children were administered the turning peg (preferred and non-preferred hand), triangle with nuts and bolts, and drawing trail III tests. According to the manual of the M-ABC2, the standard scores of the participants were calculated from the raw scores.

In this study, we were interested in examining the relationship between the variables measured by the BC task and specific fine motor skills. Therefore, we calculated the standard scores for four types of fine motor skills: preferred hand skill, non-preferred hand skill, bimanual skill, and handwriting skill, as well as the total standard score. Each standard score reflects the degree of fine motor skill at each year of age, with higher scores indicating better fine motor skills within each age group. A specifically trained and certified physical therapist administered these assessments.

### Statistical analysis

We used the chi-square test for independence to compare sex and preferred hand between age groups.

Based on the results of the Shapiro–Wilk test, the OIs in the unimanual condition (UM-OI) and the bimanual condition (BM-OI) were not normally distributed within each age group. Therefore, the Wilcoxon signed-rank test was used to compare the OI between conditions within each age group.

Since the Shapiro–Wilk test showed that the UM-OI, the BM-OI, and the bimanual coupling effect (BCE) were not normally distributed in each age group, we compared these variables between age groups using the Kruskal–Wallis test. For post-hoc analysis, we used the Mann–Whitney *U* test, and Bonferroni-corrected *p*-values were applied for multiple comparisons.

Since the Shapiro–Wilk test showed that age, UM-OI, BM-OI, and BCE were not normally distributed among all participants, we examined the correlations between age and UM-OI, BM-OI, and BCE using Spearman’s rank correlation coefficient.

Finally, partial correlation analyses using Pearson’s method were conducted to examine the relationships between the variables measured in the BC task (UM-OI, BM-OI, and BCE) and the four types of fine motor skills (preferred hand skill, non-preferred hand skill, bimanual skill, and handwriting skill), while controlling for age.

We set the significance level at *α* = 0.05 for all analyses, and we used Bonferroni’s correction to adjust for multiple comparisons. In addition, we calculated the effect size. All statistical analyses were performed using SPSS ver. 29 (IBM Corporation, Armonk, NY, United States).

## Results

Table 1 summarizes the data obtained for each age group. There were no significant differences in sex [*χ^2^*(8) = 4.623, *p* = 0.797, *φ* = 0.176], and preferred hand [*χ^2^*(8) = 9.630, *p* = 0.292, *φ* = 0.253] among the age groups.

In all age groups, BM-OI significantly increased compared to UM-OI (5-year-old, *z* = −2.073, *p* = 0.038, *r* = −0.691; 6-year-old, z = −3.459, *p* = 0.001, *r* = −0.755; 7-year-old, *z* = −3.702, *p* < 0.001, *r* = −0.849; 8-year-old, *z* = −4.286, *p* < 0.001, *r* = −0.875; 9-year-old, *z* = −3.549, *p* < 0.001, *r* = −0.837; 10-year-old, *z* = −3.408, *p* = 0.001, *r* = −0.880; 11-year-old, z = −3.621, *p* < 0.001, *r* = −0.878; 12-year-old, *z* = −2.903, *p* = 0.004, *r* = −0.838; 13-year-old, *z* = −3.408, *p* = 0.001, *r* = −0.880).

**Figure 2:**
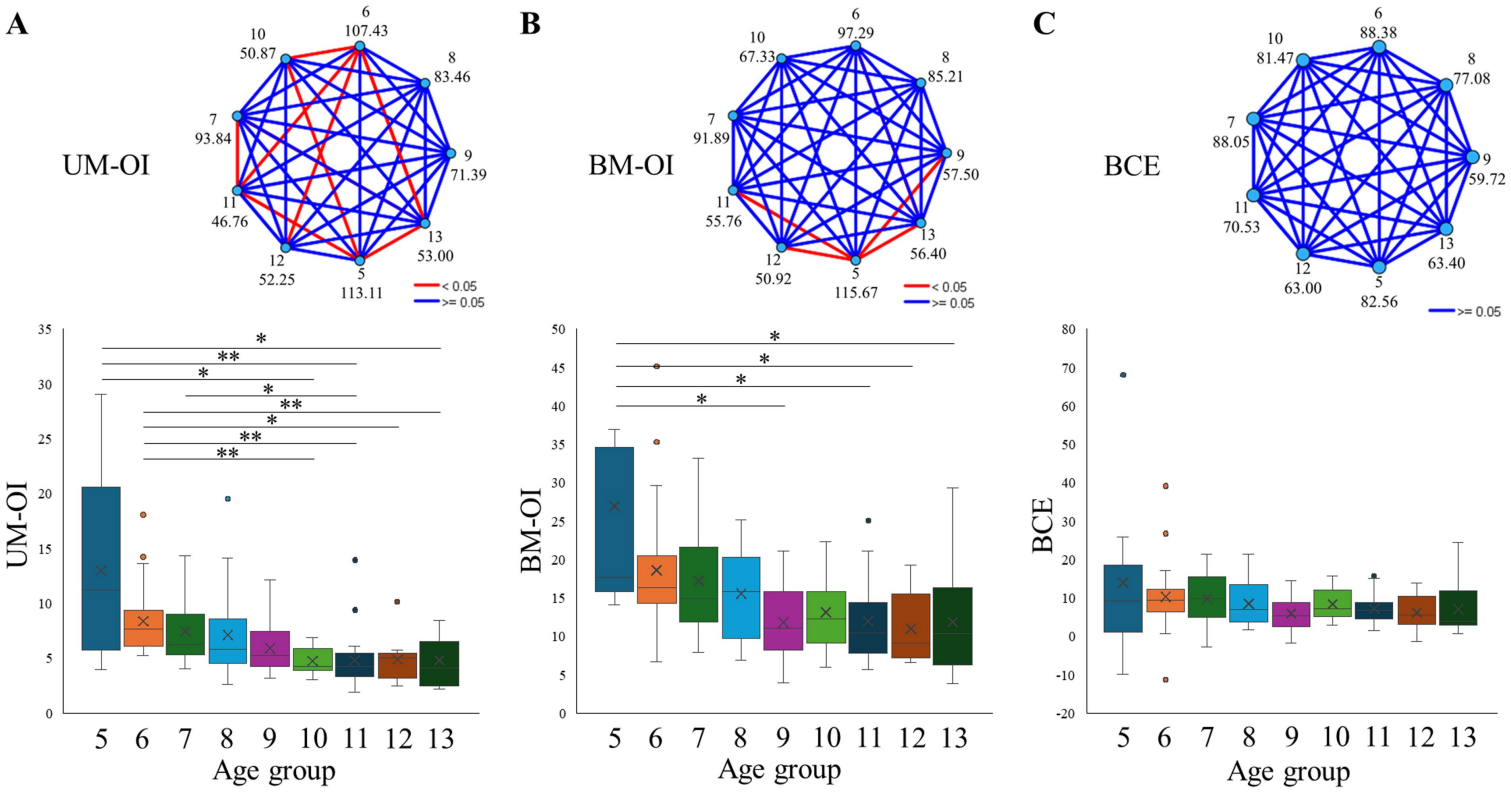
Results of the age group comparisons for the variables measured in the bimanual coupling task. **(A)** Age group comparisons of the ovalization index in the unimanual condition (UM-OI). The upper panel shows the pairwise comparisons of age groups in the Kruskal-Wallis test. Each node represents the sample average rank of a group. Blue lines indicate non-significant comparisons (adjusted *p* ≥ 0.05), while red lines indicate significant comparisons (adjusted *p* < 0.05). The lower panel presents the results of the age group comparisons. *p < 0.05, **p < 0.01. **(B)** Age group comparisons of the ovalization index in the bimanual condition (BM-OI). The upper panel shows the pairwise comparisons of age groups in the Kruskal–Wallis test. Each node represents the sample average rank of a group. Blue lines indicate non-significant comparisons (adjusted *p* ≥ 0.05), while red lines indicate significant comparisons (adjusted *p* < 0.05). The lower panel presents the results of the age group comparisons. *p < 0.05, **p < 0.01. **(C)** Age group comparisons of the bimanual coupling effect (BCE). The upper panel shows the pairwise comparisons of age groups in the Kruskal–Wallis test. Each node represents the sample average rank of a group. Blue lines indicate non-significant comparisons (adjusted *p* ≥ 0.05), while red lines indicate significant comparisons (adjusted *p* < 0.05). The lower panel presents the results of the age group comparisons. *p < 0.05, **p < 0.01.

Figure 2 shows the results of the age group comparisons for the three variables measured in the BC task (UM-OI, BM-OI, and BCE). The Kruskal–Wallis test revealed a significant difference in UM-OI among the age groups (*p* < 0.001) (Figure 2A). Multiple comparisons in post-hoc analysis showed that UM-OI significantly decreased in the 10-year-old group compared to the 5-year-old (*p* = 0.024, *r* = −0.627) and 6-year-old groups (*p* = 0.004, *r* = −0.666). The UM-OI in the 11-year-old group significantly decreased compared to the 5-year-old (*p* = 0.008, *r* = −0.576), 6-year-old (*p* = 0.001, *r* = −0.645), and 7-year-old groups (*p* = 0.042, *r* = −0.536). The UM-OI in the 12-year-old group significantly decreased compared to the 6-year-old group (*p* = 0.016, *r* = −0.684). The UM-OI in the 13-year-old group significantly decreased compared to the 5-year-old (*p* = 0.037, *r* = −0.59) and 6-year-old groups (*p* = 0.008, *r* = −0.532).

The Kruskal–Wallis test also revealed a significant difference in BM-OI among the age groups (*p* < 0.001) (Figure 2B). Multiple comparisons in post-hoc analysis showed that BM-OI in the 5-year-old group significantly increased compared to the 9-year-old (*p* = 0.037, *r* = −0.614), 11-year-old (*p* = 0.030, *r* = −0.608), 12-year-old (*p* = 0.026, *r* = −0.667), and 13-year-old groups (*p* = 0.044, *r* = −0.566).

In contrast, there was no significant difference in BCE among the age groups (*p* = 0.365) (Figure 2C).

**Figure 3:**
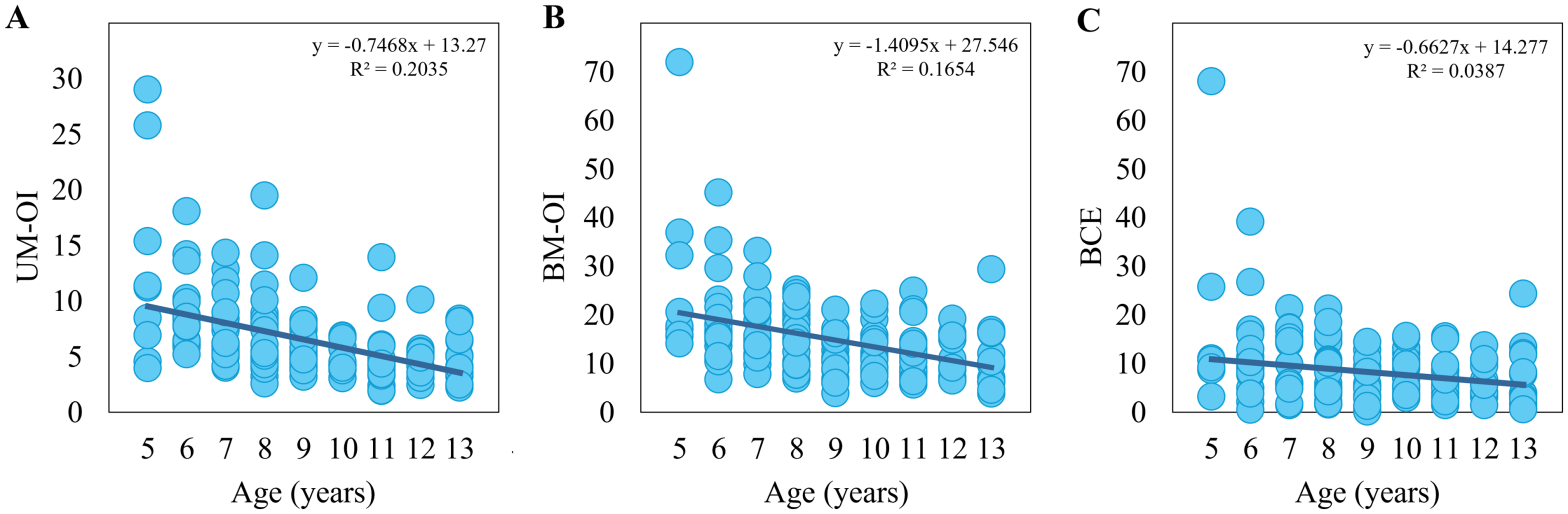
Scatter plots of age and the variables measured in the bimanual coupling task for all participants. **(A)** Correlation between age and the ovalization index in the unimanual condition (UM-OI) for all participants. **(B)** Correlation between age and the ovalization index in the bimanual condition (BM-OI) for all participants. **(C)** Correlation between age and the bimanual coupling effect (BCE) for all participants.

There were significant negative correlations between age and the variables measured in the BC task: UM-OI (*rs* = −0.510, *p* < 0.001) (Figure 3A), BM-OI (*rs* = −0.426, *p* < 0.001) (Figure 3B), and BCE (*rs* = −0.186, *p* = 0.022) (Figure 3C).

**Figure 4:**
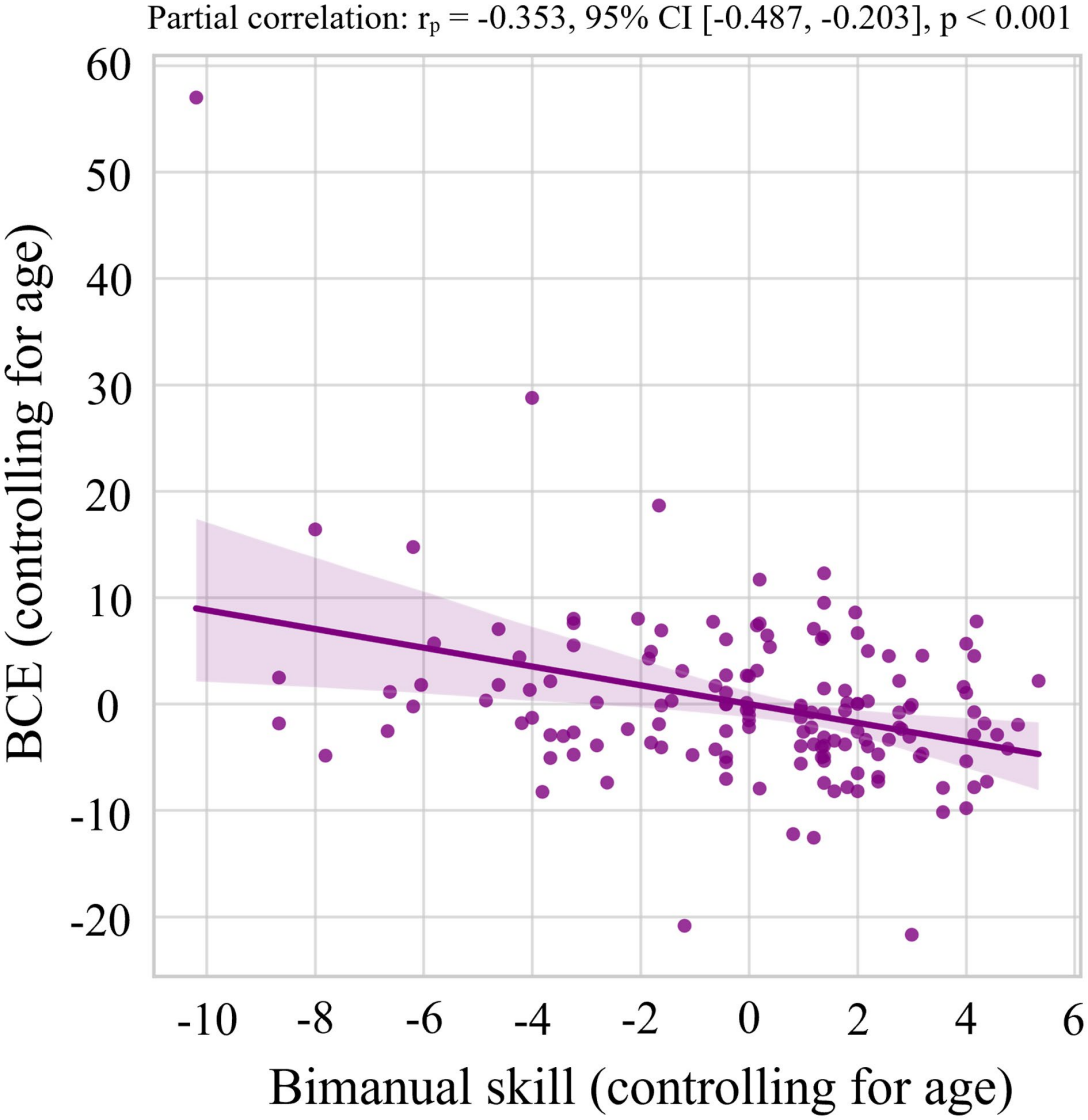
Partial correlation between the Bimanual Coupling Effect and bimanual skill, controlling for age. A significant negative partial correlation was observed between the Bimanual Coupling Effect (BCE) and bimanual skill (*rₚ* = −0.353, *p* < 0.001). That is, the higher the ability to move both hands independently and simultaneously, the higher the bimanual skill. CI, confidence interval.

Table 2 presents the results of the Pearson partial correlation analysis, controlling for age, between the three variables measured in the BC task (UM-OI, BM-OI, and BCE) and the four types of fine motor skills (preferred hand skill, non-preferred hand skill, bimanual skill, and handwriting skill). BM-OI showed significant negative partial correlations with preferred hand skill (*rₚ* = −0.262, *p* = 0.001), non-preferred hand skill (*rₚ* = −0.286, *p* < 0.001), bimanual skill (*rₚ* = −0.369, *p* < 0.001), and total fine motor skill (*rₚ* = −0.342, *p* < 0.001). Additionally, BCE showed significant negative partial correlations with non-preferred hand skill (*rₚ* = −0.263, *p* = 0.001), bimanual skill (*rₚ* = −0.353, *p* < 0.001) (Figure 4), and total fine motor skill (*rₚ* = −0.295, *p* < 0.001).

**Table 2:** Partial correlations between variables of the bimanual coupling task and four types of fine motor skills, controlling for age.

Control variable			Fine motor skills				
			Preferred hand skill	Non-preferred hand skill	Bimanual skill	Handwriting skill	Total
Age (years)	UM-OI	Partial correlation coefficient	−0.112	−0.026	−0.001	−0.096	−0.075
		*p*-value	0.175	0.757	0.992	0.245	0.366
		95% CI Lower	−0.269	−0.187	−0.163	−0.254	−0.234
		95% CI Upper	0.051	0.136	0.161	0.067	0.088
	BM-OI	Partial correlation coefficient	−0.262	−0.286	−0.369	0.024	−0.342
		*p*-value	0.001	<0.001	<0.001	0.772	<0.001
		95% CI Lower	−0.407	−0.428	−0.501	−0.138	−0.477
		95% CI Upper	−0.105	−0.130	−0.220	0.185	−0.191
	BCE	Partial correlation coefficient	−0.201	−0.263	−0.353	0.066	−0.295
		*p*-value	0.014	0.001	<0.001	0.426	<0.001
		95% CI Lower	−0.351	−0.408	−0.487	−0.097	−0.436
		95% CI Upper	−0.040	−0.106	−0.203	0.225	−0.140

BCE, bimanual coupling effect; BM-OI, the ovalization index (OI) in the bimanual condition (BM); CI, confidence interval; UM-OI, the ovalization index (OI) in the unimanual condition (UM).

## Discussion

The present study investigated the developmental trajectory of the bimanual coupling effect (BCE) and its association with fine motor skills in children aged 5 to 13 years. Across all age groups, a significant increase in ovalization index (OI) from the unimanual (UM) to the bimanual (BM) condition was observed, indicating the presence of BCE. Age group comparisons revealed that both UM-OI and BM-OI significantly decreased with increasing age. Furthermore, correlation analyses demonstrated significant negative associations between age and UM-OI, BM-OI, and BCE. These findings suggest that not only unimanual motor performance, as reflected by UM-OI, but also the ability to control both hands independently and simultaneously, as indicated by BM-OI and BCE, improves with age from 5 to 13 years.

In early infancy, children can only perform simple symmetrical bimanual movements. However, by around 2 years of age, more complex asymmetric bimanual actions begin to emerge (Fagard and Jacquet, 1989). Developmental studies focusing on the contrast between symmetrical and asymmetrical bimanual coordination have shown that seven-year-olds perform symmetrical movements more accurately than asymmetrical ones, but this difference diminishes significantly between the ages of 8 and 10 (Fagard and Pezé, 1992; Fagard et al., 2001; Fagard, 1987). Indeed, symmetrical or mirror movements are frequently observed in young children (Utley and Steenbergen, 2006), but they dramatically decrease and stabilize by around 8.5 years, becoming rare by age 9 (Cohen et al., 1967; Lazarus and Todor, 1987). In addition, the speed and accuracy of bimanual coordination tasks requiring each hand to control movement along a different axis, such as drawing along *x*- and *y*-coordinates, have been shown to improve significantly between ages 6 and 13 (Marion et al., 2003; Steese-Seda et al., 1995).

Neuroimaging studies have revealed that asymmetric bimanual movements, such as those required in the BM condition of the BC task, activate the fronto-parietal network—including the supplementary motor area, premotor cortex, superior parietal lobule, and inferior parietal lobule—more strongly than do symmetrical bimanual movements (Garbarini et al., 2014; Iester et al., 2025; Sadato et al., 1997; Wenderoth et al., 2004). Moreover, the fronto-parietal network involved in bimanual control continues to mature between ages 6 and 10 and reaches full maturation during adolescence (Casey et al., 2005; Gogtay et al., 2004).

In addition, the BC task requires dual attention control, as it involves simultaneously drawing straight lines with one hand and circles with the other. This necessitates high-level cognitive control centered in the prefrontal cortex, particularly executive functions and working memory. These functions are known to be still immature at approximately 6 years of age and are reported to stabilize around the age of 8 (Brocki and Bohlin, 2004; Frick and Möhring, 2016).

Furthermore, the age-related improvement in BCE observed in this study may be explained by the structural maturation of transcallosal motor fibers (TCMFs) and the accompanying enhancement of interhemispheric inhibition (IHI). Indeed, Kwon et al. (2014) demonstrated that TCMFs originating from the corticospinal tract begin to appear after age 6 and significantly increase in prevalence around age 10, suggesting a marked structural development of interhemispheric motor communication during middle childhood. Thus, the age-related decrease in BCE observed in this study may reflect enhanced capacity to suppress involuntary mirror movements as a result of the maturation of IHI mechanisms.

In line with these findings, the present study provides further evidence that the ability to perform simultaneous and independent bimanual movements improves throughout middle childhood. Notably, although age-related improvements in UM-OI and BM-OI were demonstrated both through age group comparisons and correlation analyses, age-related improvement in BCE was observed only in the correlation analyses. This suggests that the development of the ability to control each hand independently during simultaneous movement progresses gradually rather than abruptly between ages 5 and 13. One possible explanation for the lack of significant group differences in BCE is that changes in BCE across adjacent age groups may be subtle, and the high inter-individual variability combined with limited group sizes may have reduced the statistical power to detect between-group differences. In contrast, correlation analyses treat age as a continuous variable and are more sensitive to detecting linear developmental trends across a wide age range. Therefore, we considered the correlation analysis to be a more appropriate and powerful method for capturing the developmental trajectory of BCE in this study.

Another aim of the present study was to examine which specific fine motor skill(s), among four measured using the M-ABC2, are associated with the ability to perform independent simultaneous bimanual movements, as indicated by BM-OI and BCE. Interestingly, although UM-OI reflects the degree of ovalization during repetitive straight-line drawing with the preferred hand, no significant association was found between UM-OI and preferred hand skill. In contrast, BM-OI was significantly associated with both preferred and non-preferred hand skills, and BCE was significantly associated with non-preferred hand skill. These results suggest that the ability to draw straight lines with the preferred hand while simultaneously performing a conflicting circular movement with the non-preferred hand, as indexed by BM-OI, may reflect a higher level of skill in independently coordinating each hand. That is, greater independence between the hands is likely to be achieved when both the preferred and non-preferred hands are sufficiently proficient. Similarly, the significant association between BCE and non-preferred hand skill implies that greater control over the non-preferred hand may contribute to reducing the interference effect between hands during simultaneous movements.

Most importantly, partial correlation analyses controlling for age revealed that among the four types of fine motor skills assessed, both BM-OI and BCE were significantly associated with bimanual skills. This indicates a meaningful relationship between the ability to independently control both hands and overall bimanual coordination skill. However, this result is not surprising. In this study, the bimanual skills subtest of the M-ABC2 included age-appropriate tasks: the threading beads task for ages 5–6, the threading lace task for ages 7–10, and the triangle with nuts and bolts task for ages 11–13. Each of these tasks requires both the preferred and non-preferred hands to perform different yet complementary movements toward a shared goal. Thus, the finding that better performance in the BC task is associated with higher bimanual skill appears to be highly valid.

Moreover, this result supports the utility of the BC task as a method for evaluating bimanual coordination skills. While M-ABC2 is a well-established assessment tool for fine motor abilities, other standardized tests such as the Bruininks–Oseretsky Test of Motor Proficiency, Second Edition (BOT-2; Bruininks and Bruininks, 2005) and the Peabody Developmental Motor Scales, Second Edition (PDMS-2; Folio and Fewell, 2000) are also frequently used. These tests evaluate various fine motor skills—such as cutting along a line with scissors, copying shapes, sorting cards, stacking blocks, or open the lid of a container—and require different sets of materials depending on the age of the child. In contrast, the present findings suggest that the BC task may serve as a practical alternative for assessing bimanual coordination skills across a wide age range, without the need for task variation or extensive material preparation.

This study has several limitations. First, it was not possible to equalize the sample size across age groups. This may have been a primary factor contributing to the lack of statistically significant differences in BCE across age groups. Future studies should aim to balance the sample size across age groups and increase the overall number of participants. Second, in the present study, we did not measure the repetitive circular trajectories performed by the non-preferred hand during the BM condition of the BC task. Simultaneous measurement of both the vertical line trajectories of the preferred hand and the circular trajectories of the non-preferred hand may allow for a more detailed assessment of the BCE. Third, the findings of this study suggest that the BC task has potential as a quantitative measure of bimanual coordination skills. However, to further validate this potential, future research should incorporate additional standardized assessments of children’s fine motor skills beyond the M-ABC2 (Henderson et al., 2007), such as the BOT-2 (Bruininks and Bruininks, 2005) and the PDMS-2 (Folio and Fewell, 2000). Lastly, while this study revealed developmental changes in the ability to perform independent simultaneous bimanual movements during childhood, it did not directly examine the relationship between these changes and the underlying neural mechanisms —such as the maturation of the fronto-parietal network and the increase in IHI—that are assumed to support this ability. Future research addressing this question would contribute to a more comprehensive understanding of the development of upper-limb motor control in childhood.

## Conclusion

This study behaviorally demonstrated that the bimanual coupling effect (BCE)—as measured by the BC task, and reflecting the ability to perform independent simultaneous bimanual movements—improves between the ages of 5 and 13. It also revealed a significant relationship between this ability and bimanual coordination skills. Future studies that take into account the limitations of the present research are needed to further investigate the developmental changes in both the ability to move both hands independently and its underlying neural mechanisms, as well as to evaluate the potential of the BC task as a standardized assessment tool for bimanual coordination skills.

## Data Availability

The raw data supporting the conclusions of this article will be made available by the authors, without undue reservation.
